# Highly comparative time series analysis of oxygen saturation and heart rate to predict respiratory outcomes in extremely preterm infants

**DOI:** 10.1088/1361-6579/ad4e91

**Published:** 2024-06-03

**Authors:** Jiaxing Qiu, Juliann M Di Fiore, Narayanan Krishnamurthi, Premananda Indic, John L Carroll, Nelson Claure, James S Kemp, Phyllis A Dennery, Namasivayam Ambalavanan, Debra E Weese-Mayer, Anna Maria Hibbs, Richard J Martin, Eduardo Bancalari, Aaron Hamvas, J Randall Moorman, Douglas E Lake

**Affiliations:** 1 Department of Medicine, Division of Cardiology, University of Virginia School of Medicine, Charlottesville, VA, United States of America; 2 Department of Pediatrics, Case Western Reserve University School of Medicine, University Hospitals Rainbow Babies and Children’s Hospital, Cleveland, OH, United States of America; 3 Department of Pediatrics, Division of Autonomic Medicine, Northwestern University Feinberg School of Medicine, Chicago, IL, United States of America; 4 Department of Pediatrics, Division of Neonatology, University of Miami Miller School of Medicine, Miami, FL, United States of America; 5 Department of Pediatrics, Division of Neonatology, University of Alabama at Birmingham, Birmingham, AL, United States of America; 6 Department of Pediatrics, Brown University School of Medicine, Department of Pediatrics, Providence, RI, United States of America; 7 Department of Electrical Engineering, University of Texas at Tyler, Tyler, TX, United States of America; 8 Ann and Robert H. Lurie Children’s Hospital and Northwestern University Department of Pediatrics, Chicago, IL, United States of America; 9 Department of Pediatrics, Division of Pediatric Pulmonology, Washington University School of Medicine, St. Louis, MO, United States of America; 10 Department of Pediatrics, University of Arkansas for Medical Sciences and Arkansas Children’s Hospital, Little Rock, AR, United States of America

**Keywords:** highly comparative time series analysis, preterm infants, intermittent hypoxemia, predictive models

## Abstract

*Objective.* Highly comparative time series analysis (HCTSA) is a novel approach involving massive feature extraction using publicly available code from many disciplines. The Prematurity-Related Ventilatory Control (Pre-Vent) observational multicenter prospective study collected bedside monitor data from ${\gt}700$ extremely preterm infants to identify physiologic features that predict respiratory outcomes. *Approach*. We calculated a subset of 33 HCTSA features on ${\gt}7$ M 10 min windows of oxygen saturation (SPO2) and heart rate (HR) from the Pre-Vent cohort to quantify predictive performance. This subset included representatives previously identified using unsupervised clustering on ${\gt}3500$ HCTSA algorithms. We hypothesized that the best HCTSA algorithms would compare favorably to optimal PreVent physiologic predictor IH90_DPE (duration per event of intermittent hypoxemia events below 90%). *Main Results.* The top HCTSA features were from a cluster of algorithms associated with the autocorrelation of SPO2 time series and identified low frequency patterns of desaturation as high risk. These features had comparable performance to and were highly correlated with IH90_DPE but perhaps measure the physiologic status of an infant in a more robust way that warrants further investigation. The top HR HCTSA features were symbolic transformation measures that had previously been identified as strong predictors of neonatal mortality. HR metrics were only important predictors at early days of life which was likely due to the larger proportion of infants whose outcome was death by any cause. A simple HCTSA model using 3 top features outperformed IH90_DPE at day of life 7 (.778 versus .729) but was essentially equivalent at day of life 28 (.849 versus .850). *Significance*. These results validated the utility of a representative HCTSA approach but also provides additional evidence supporting IH90_DPE as an optimal predictor of respiratory outcomes.

## 1. Introduction

### 1.1. Highly comparative time series analysis (HCTSA)

Analysis of heart rate (HR) and oxygen saturation (SPO2 or more precisely *SpO*_2_) vital sign time series during the stay of infants in the neonatal intensive care unit (NICU) has been shown to be useful in predicting unfavorable outcomes. This is part of larger efforts to improve care of infants using big data analytics (Cole 2021, Vesoulis *et al* 2023). Notably, models for predicting sepsis have not only been developed (Griffin *et al* 2003, Fairchild *et al* 2017, Kausch 2023) but implemented in the NICU and shown to improve outcomes in a large clinical trial (Moorman 2011, Fairchild 2013). Models have also been developed for other adverse outcomes during NICU stay including death (Sullivan *et al* 2016, 2018, Niestroy *et al* 2022), bronchopulmonary dysplasia (BPD) (Raffay *et al* 2019, Gentle *et al* 2023, Ramanand *et al* 2023), and retinopathy of prematurity (Di Fiore *et al* 2010, 2012). Vital sign data during an infant’s stay has also been shown to predict long-term outcomes of cognitive impairment (Poets 2015), cerebral palsy (Letzkus *et al* 2022), and autism (Blackard *et al* 2021).

There have been many prediction models developed for the respiratory outcome BPD and tools are available to estimate risk at the bedside using demographic and clinical features (but no vital sign features). A recent review of and over 64 studies and 53 models in 275 000 infants concluded that published BPD models were mostly outdated, single center and lack external validation (Kwok *et al* 2023). A notable exception to this comes from a large study for predicting BPD that was recently conducted by the National Institute of Child Health and Human Development (NICHD) Neonatal Research Network (Greenberg 2022) on 9181 infants in NICU from 2011 to 2017. The resulting BPD risk estimator is available online and has been internally and externally validated (https://neonatal.rti.org/index.cfm).

Highly comparative time-series analysis (HCTSA) developed by Fulcher *et al* (2013) is a novel method that naturally applies to HR and SPO2 data. series algorithms with various sets of parameter values. The core concept involves using numerous time-series algorithms with a wide-ranging set of parameter values to extract a massive number of features to associate with some target outcome. They examined over 35 000 real-world and model-generated time series with more than 7000 time-series analysis algorithms developed from a wide variety of disciplines. The MATLAB code to implement these HCTSA algorithms is publicly available at https://github.com/benfulcher/hctsa. The goal of using HCTSA is not necessarily to develop an optimal predictive model restricted to these algorithms. Here we use HCTSA as a tool to identify new types of algorithms for future more traditional development with sufficient understanding to be useful for clinicians.

Recently, Niestroy *et al* (2022) applied ${\gt}3500$ HCTSA algorithms to ${\gt}17$M 10 min windows of HR and oxygen saturation (SPO2) vital sign data collected from bedside monitors (displayed every two seconds) from 6000 infants at the University of Virginia NICU from 2009 to 2019. The data (including all HR and SPO2 time series) is publicly available at Niestroy *et al* (2021). In an effort to reduce the high dimensionality of the data, a random subset of ${\gt}120$ K daily results was then used for unsupervised *k*-medoids clustering based on distance metric of mutual information. With *k* = 20 clusters, 81% of the variance of full data was explained and identified 20 central algorithms or medoids. A medoid is defined to be a point in the cluster from which the sum of distances to other data points in the cluster is minimal.

Utilizing these clusters, they found that HCTSA algorithms can discover novel patterns associated with neonatal mortality in the next 7 days. Notably, models based solely on the cluster centers performed comparably to those considering the full feature set. This lead to a hypothesis that identifying algorithms by unsupervised clustering could capture most of the predictive information in NICU vital sign data and that motivates this work.

Based on this hypothesis, a subset of HCTSA algorithms (including at least one from each of the 20 clusters) was implemented using the publicly available code as part of the Batch Algorithm Processor (BAP) software package developed for comprehensive analysis of bedside waveform and vital sign time series data. In addition, the maximum and minimum cross-correlation of HR and SPO2 at lags up to 30 s were calculated which have been shown to be associated with sepsis, apnea and periodic breathing (Fairchild and Lake 2018) giving a total of 33 vital sign features calculated. The BAP software including extensive documentation is available at https://github.com/UVA-CAMA/BatchAlgorithmProcessor.

### 1.2. PreVent study

The Prematurity-Related Ventilatory Control (Pre-Vent) study was of a cohort of ${\gt}700$ extremely premature infants (gestational age less than 29 weeks) across 5 NICU sites with the hypothesis that physiologic features of ventilatory control extracted from bedside monitoring data can predict unfavorable respiratory outcomes at 40 weeks post-menstrual age (PMA) (Dennery 2019). The BAP was developed for Pre-Vent and used to extract a large number of physiologic (including HCTSA) features. The software was run at each of the sites remotely in a separate but uniform way while keeping raw data stored locally. A result of this processing was 33 features calculated on 7.8M 10 min windows of HR and SPO2 data.

In the primary analysis of the Pre-Vent study, optimal logistic regression models were developed for predicting respiratory outcomes at varying days of life during the NICU stay and identified important risk factors for both physiologic and clinical models (Ambalavanan 2023). Physiologic models included metrics calculated from bedside waveforms (ECG and chest impedance) and vital signs (HR and SPO2). Notably, the duration per event (DPE) of intermittent hypoxemia events with SPO2$ < 90\%$ (IH90) was the most significant individual physiologic factor for predicting the primary unfavorable respiratory outcome. At day of life 28, performance of IH90_DPE as an individual predictor performed similarly to optimal models with demographic, respiratory support and other clinical features. However, IH and physiologic models in general did not perform as well earlier in the NICU stay (day 7) where identification of higher-risk infants would have more impact.

### 1.3. Intermittent hypoxemia

Intermittent hypoxemia (IH) in the NICU has been widely studied (Ramirez *et al* 2023, Dormishian *et al* 2023a, 2023b). Definitions of IH events require fixed parameters including SPO2 threshold (e.g. 80% or 90%), min/max duration, and possibly joining rules for nearby events. These definitions can be somewhat arbitrary and susceptible to varying hospital protocols (e.g. target SPO2 ranges) or clinical practice. IH events can also be sensitive to the averaging time of the pulse oximeter which can vary across vendor and NICU. Published physiological models relying on threshold-based events may not prove to be robust in universal and evolving applications. Using advanced time-series metrics to predict neonatal respiratory outcomes without relying on clinical definitions have not yet been fully studied.

## 2. Methods

### 2.1. Study population

The study population consisted of the 717 extremely premature infants prospectively enrolled in Pre-Vent study and with respiratory outcome determined (Ambalavanan 2023). Analysis was restricted to days where sufficient HR and SPO2 monitor data was available (at least 12 hours of each) which represented over 80 percent of the study period.

The Pre-Vent study predefined 5 mutually exclusive respiratory outcome categories which in order of decreased severity are:

(i) **Death:** prior to 40 weeks PMA
(ii) **Severe:** invasive mechanical ventilation (IMV) at 40 weeks PMA
(iii) **Moderate:** need for positive pressure at 40 weeks PMA
(iv) **Mild:** respiratory medications, oxygen or other respiratory support either inpatient at 40 weeks PMA or at discharge prior to 40 weeks PMA
(v) **Favorable:** none of the above

The primary outcome of the Pre-Vent study was an unfavorable outcome consisting of any of the first 4 outcomes (i.e. not favorable). Three additional binary outcomes analyzed here in order of increase severity are moderate or severe or death, severe or death and death. For each outcome, the entire cohort was used to compare having vs. not having outcome.

**Figure 1.**
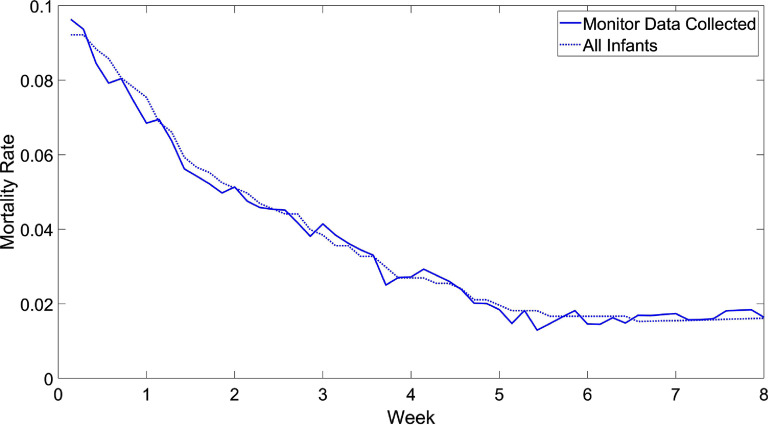
Mortality rate by week of life. For each time point, the solid line shows the mortality rate in patients with monitoring data collected and the dotted line shows the mortality rate in all infants in study.

Predictive performance of the time series features were evaluated at day of life 7, 14, and 28 which follows the approach of the PreVent analysis (Ambalavanan 2023). Table 1 shows a breakdown of the infants by binary outcomes at these time points. A particular focus was predicting an unfavorable outcome at day 7, which consisted of 584 infants and an event rate of 265/584 = 45.4%. An important aspect of the analysis to consider is the evolving distribution of the outcomes by day of life. Figure 1 shows how the mortality rate of surviving infants is significantly reduced by week of life. Since death outcomes include those of all causes and not necessarily related to respiratory failure, analyzing outcomes later in the NICU stay are therefore more directly associated with control of breathing. Also note in figure 1 that there is minimal difference in mortality rate between those with monitor data and entire population suggesting there is no significant selection bias in the analysis.

**Table 1.** Sample size and distribution of binary respiratory outcome at day 7, 14, and 28 for infants with monitor data collected.

	Day 7N = 584	Day 14N = 584	Day 28N = 551
Death	40 (6.8%)	30 (5.1%)	15 (2.7%)
Severe\death	80 (13.7%)	71 (12.2%)	40 (10.0%)
Severe\Death\moderate	121 (20.7%)	110 (18.8%)	92 (16.7%)
Unfavorable	265 (45.4%)	263 (45.0%)	228 (41.4%)
Favorable	319 (54.6%)	321 (55.0%)	323 (58.6%)

### 2.2. Time series features

The 33 new HR and SPO2 HCTSA features are described in table 2 along with which of the 20 clusters they belonged to and a general description of the type of algorithms in the cluster. The number of the clusters are ordered in way so that nearby clusters tend to be more related. Table A1 provides more details on the exact parameters and the publicly available Matlab code used for each feature. For evaluation purposes, the features were summarized as the daily median of the every 10 minute results.

**Table 2.** Time-series features implemented in Batch Algorithm Processor (BAP).

Feature	Cluster	Cluster name	Cluster descripton
hr_meanhr_avgthresh hr_corrmean	1	hr_mean	HR mean

hr_stdhr_cv	2	hr_std	HR standard deviation

hr_max	3	hr_max	HR maximum

hr_min	4	hr_min	HR minimum

hr_kurthr_skew	5	hr_skew	HR skewness

hr_symautocorr	6	hr_autocorr	HR autocorrelation

hr_surprisehr_entropy	7	hr_entropy	HR entropies

hr_wavelet	8	hr_wavelet	HR wavelet decomposition

hr_probincreaseshr_entropy_diff	9	hr_symbolic	HR symbolic transforms

sp_std	10	sp_std	SPO2 standard deviation

sp_meansp_avgthres sp_corrmean	11	sp_mean	SPO2 mean

hr_derivative	12	hr_derivative	HR *n*th derivative

sp_min	13	sp_min	SPO2 minimum

sp_skewsp_kurt sp_mean_trim	14	sp_median	SPO2 median

sp_wavelet	15	sp_misc	SPO2 miscellaneous

sp_symeigen	16	sp_symbolic	SPO2 symbolic transforms

sp_walksp_autocorr	17	sp_autocorr	SPO2 autocorrelation

sp_symentropy	18	sp_entropy	SPO2 entropies

sp_symbin	19	sp_binary	SPO2 binary symbolic transforms

sp_symiqr	20	sp_more	SPO2 more symbolic transforms

xc_max			Maximum cross-correlation
xc_min			Minimum cross-correlation

The daily HCTSA results were merged with the physiological features measured on HR and SPO2 as part of PreVent study. Using the BAP, the following physiologic events were extracted from bedside sign monitor data:

(i) IH80: desaturation events with SPO2 $ < 80\%$ for 10–300 s
(ii) IH90: desaturation events with SPO2 $ < 90\%$ for 10–300 s
(iii) Brady80: bradycardia events with HR $ < 80$ beats per minute for 5 or more s

These IH duration limits come from Di Fiore *et al* (2012) but do vary slightly across publications. For each of these 3 events, daily features were quantified as count of events per day (count), total daily duration in events (dur) measured in minutes per day, and average DPE measured in seconds per event. These 3 quantities are not independent and have relationship dpe = 60×dur/count. These 9 clinically defined features gave a total of 42 candidate HR and SPO2 metrics for the analysis.

### 2.3. Feature evaluation

We evaluated the performance of each feature based on the area under the receiver operating curve (AUC) at various days of life for all 4 binary outcomes. The AUC for predicting the primary unfavorable outcome at day 7 was used to rank the features. Signature of risk curves showing estimates of the probability of the unfavorable outcome at day 7 as a function of the daily median value of each variable were made using logistic regression model including a cubic spline transformation with 3 knots to account for possible non-linearity. The top performers were then analyzed further by looking at a range of examples stratified by percentile to better understand what the algorithm was measuring and, in particular, its correlation with IH events. The trajectories of the performance of these new metrics was also analyzed for the first 8 weeks of life.

For logistic regression models, AUC was measured using cross-validation where each fold consisted of infants clustered by pregnancy so that an infant’s sibling was not used to predict its outcome. Variable importance was quantified by the drop in cross-validated AUC when the variable was removed from the model.

### 2.4. Demographic and clinical risk factors

Detailed demographic information about the Pre-Vent cohort can be found in Ambalavanan (2023). The sex of the infants in the study were equally represented (51% male) and the average birth weight and gestational age were 871 grams and 26.4 weeks respectively. There is also a comprehensive list of clinical risk factors for predicting respiratory outcomes at various time points. Based on this, the following were identified as the major risk factors for unfavorable respiratory outcomes and included in the presented results for comparison purposes.

(i) BW: birthweight in grams
(ii) GA: gestational age in weeks
(iii) IMV: daily need for invasive mechanical ventilation
(iv) FIO2 (or *FiO*_2_): fraction of inspired oxygen closest to noon (0.21 if on room air)

Notably, no major differences in the results by sex were found.

## 3. Results

### 3.1. Top features for predicting unfavorable outcome

Table 3 summarizes the top individual performers in order of highest AUC for predicting primary unfavorable outcome at day 7. Results and ranks (out of 42) for days 14 and 28 are also included. Comprehensive results for all features and each of the outcomes for days 7, 14 and 28 is provided in tables A2–A4. Performance of the major clinical risk factors are also provided in table A6 for comparison. In addition, correlation of the 42 features with clinical risk factors at day 7 are provided in table A5. Not surprisingly, there is a high correlation between time series features that predict outcomes well and birthweight, gestational age, IMV and FIO2.

**Table 3.** Top performers for predicting primary unfavorable respiratory outcome and respective ranks (out of 42) at Day 7, 14 and 28.

Feature	Cluster	Day 7	Rank	Day 14	Rank	Day 28	Rank
sp_walk	17	0.761	1	0.797	1	0.851	1
sp_autocorr	17	0.740	2	0.782	3	0.841	3
sp_avgthres	11	0.735	3	0.741	7	0.784	8
sp_mean	11	0.731	4	0.732	8	0.772	10
sp_corrmean	11	0.731	5	0.731	9	0.769	11
ih90_dur		0.730	6	0.742	6	0.797	6
ih90_dpe		0.729	7	0.792	2	0.850	2
sp_mean_trim	14	0.725	8	0.720	11	0.765	12
sp_symeigen	16	0.718	9	0.749	5	0.814	5
sp_min	13	0.703	10	0.666	21	0.655	24
sp_kurt	14	0.698	11	0.750	4	0.817	4
ih90_count		0.696	12	0.663	22	0.691	16
hr_entropy_diff	9	0.689	13	0.704	13	0.688	17
hr_entropy	7	0.675	14	0.647	25	0.635	27
hr_probincreases	9	0.672	15	0.686	14	0.679	19
ih80_dpe		0.672	16	0.724	10	0.795	7
ih80_dur		0.671	17	0.706	12	0.756	13
sp_std	10	0.665	18	0.653	24	0.675	21
ih80_count		0.652	19	0.675	17	0.714	14
hr_wavelet	8	0.626	20	0.653	23	0.645	26

**Figure 2.**
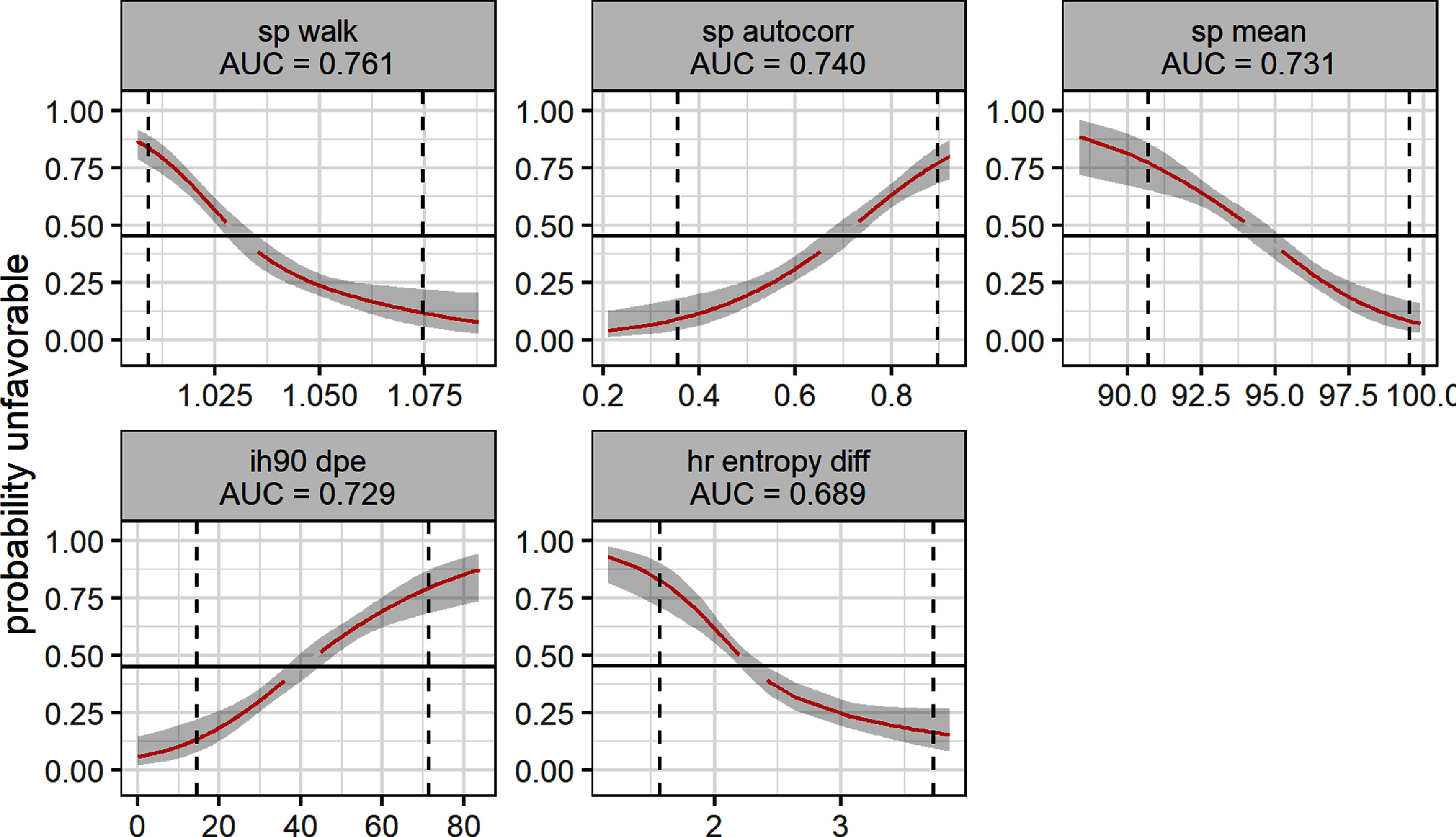
Individual signature of risk for unfavorable outcome for top predictors at day 7 including those used in multivariate HCTSA model. The gray area indicates the 95% confidence intervals. The black line represents the event rate at day 7 which from table 1 is 0.454. The red line indicates where the confidence intervals do not include this null value. The dotted lines represent the limits of the central 95% of the distribution of daily values.

Based on table 3, figure 2 shows signature of risk curves for 5 of the top predictors of an unfavorable outcome at day 7. These are discussed in more detail below and include the 3 features used in multivariate HCTSA only model.

### 3.2. Top SPO2 HCTSA features

The top HCTSA feature for predicting respiratory outcomes was sp_walk which is a time series metric inspired by physics. The feature is based on comparing the signal’s standard deviation to that of a simulated hypothetical particle (or ‘walker’) that moves in response to values of the time series at each point. As described in the Matlab code, the walker moves as if it had inertia (based on its mass which is an input parameter) from the previous time step so that it ‘wants’ to move the same amount and the original time series acts as a force changing its motion. SPO2 time series that behave very much like this simulated walk have lower ratios close to 1 which indicate higher risk of unfavorable outcomes.

This algorithm is part of a cluster of algorithms associated with the autocorrelation of the SPO2 time series and includes the central algorithm and second best performer sp_autocorr. Autocorrelation is a standard time domain tool to evaluate, among other things, the frequency content of the signal and sp_autocorr has some advantages over sp_walk in terms of interpretability. The specific parameters for sp_autocorr was the correlation of SPO2 with a delayed copy of itself at a lag of 4 samples or 8 s. High values of sp_autocorr are associated with unfavorable outcomes and occur in signals that have some variability over a 10 min window but do not have high frequency variations locally (over an 8 second window). Looking more closely at other lags and metrics associated with the autocorrelation of the SPO2 is a potentially promising area for future work. Both sp_walk and sp_autocorr were highly correlated with IH90 DPE (−.77 and .76 respectively) and are fundamentally measuring the same physiological status of the infant but perhaps in a more robust way.

**Figure 3.**
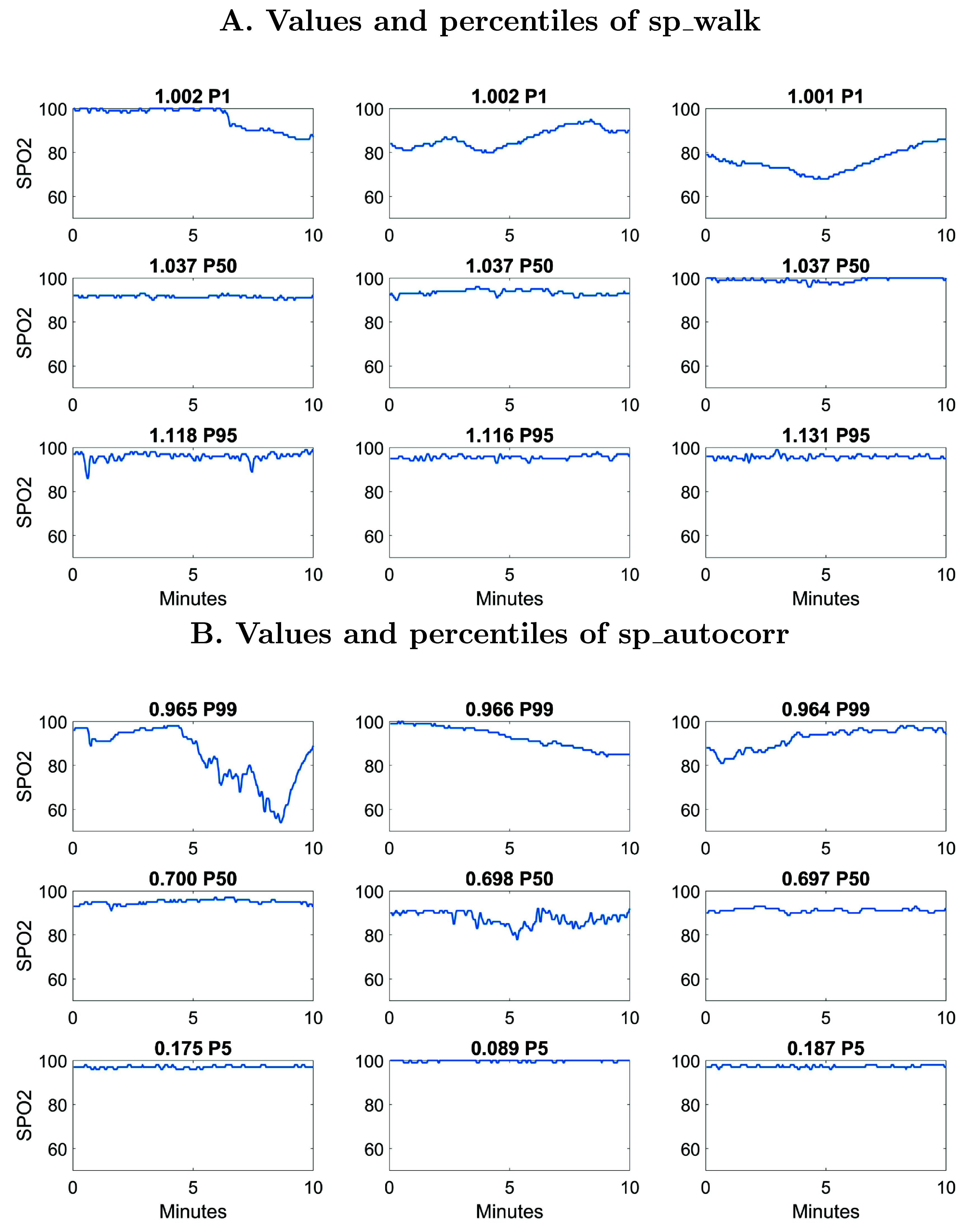
Examples of 10 min SPO2 time series on day 7 for twp top HCTSA features sp_walk and sp_autocorr. The title includes feature value and its percentile, e.g. P5 = 5th percentile of distribution. Additional feature values are provided in table A7 for comparison.

Figure 3 shows examples of 10 min SPO2 time series from extremely premature infants on day of life 7 from publicly available dataset (Niestroy *et al* 2021). Figures 3(A) and (B) shows the values and percentiles for sp_walk and sp_autocorr respectively. The top row of each figure shows higher risk examples which is low values for sp_walk and high values for sp_autocorr. These records are generally associated with persistent low-frequency patterns with desaturations that include long duration IH90 and IH80 events. The middle and bottom rows of these examples show median and percentiles associated with low risk. These exhibit some interesting patterns but not in a way that has consistent clinical interpretation like with high risk examples. Future work beyond the scope of this paper is needed to understand if the presence of subtle high-frequency variability in SPO2 is clinically meaningful.

Other top SPO2 features include sp_mean and other measures from this cluster. None of these other features outperforms sp_mean by an amount that would likely justify deviating from using a simple measure that is clinically easy to understand. Another SPO2 metric that performed well at days 14 and 28 was kurtosis. The mean and kurtosis are part of a larger group of SPO2 features that characterize a daily pulse oxygen histogram or profile. These histograms are used as a clinical tool in the NICU to evaluate target range management (Borenstein-Levin *et al* 2020, Gentle *et al* 2020, Goetz *et al* 2022). Developing predictive models based directly on these daily SPO2 histograms is a promising area for future work. From an HCTSA perspective, this would correspond to restricting analysis to metrics like moments and percentiles that do not depend on the order of the time series values.

### 3.3. Top HR HCTSA features

The top three HR features were symbolic transformation metrics associated with discretizing the HR record into sequence of either 2 (binary) or 3 symbols that are also good measures of low HR variability associated with high risk of death. The best algorithm, hr_entropy_diff, specifically first quantizes the successive HR differences into 3 symbols roughly representing increasing, same and decreasing. It then looks at the distribution of all $3^4 = 81$ possible patterns of length 4 and calculates the Shannon entropy. Low values of hr_entropy_diff are associated with large proportion of unchanging HR during 10 min window. The second best algorithm, hr_entropy, is the exact same algorithm applied to raw signal without first taking differences. The third best HR algorithm is hr_probincreases which was previously identified as best individual predictor of mortality in next week in the NICU (Niestroy *et al* 2022). This measure transforms the HR record into binary sequence of either up (u) or down (d) based on whether HR increases or not (HR staying same is considered down). The hr_probincreases is the probability of the uu pattern and low values indicate high risk of mortality.

All three of these measures are related to HR fragmentation metrics introduced recently (Costa *et al* 2017a, 2017b) and shown to be predictive of long-term survival in large study of 3000 24 hour Holter recordings from patients of all ages (Lensen *et al* 2020). A lesson learned from HCTSA is that measures that convert the HR to a simple binary time series and look for runs of ones of lengths 1, 2, 3, $\ldots$ can quantify heart-rate variability and are good candidates to predict adverse neonatal outcomes in a way clinicians can easily understand.

### 3.4. Trajectories of predictive performance

Values of the AUC for sp_walk and IH90_DPE at days 7, 14 and 28 are summarized in table 4. Based on the individual results discussed above, a simple HCTSA model using 3 fixed features sp_walk, sp_mean, and hr_entropy_diff was developed at each day of life and also included. These final features were chosen based on the cross-validated AUC at day 7. The criteria was that removing any of the features from the model reduced the AUC by more than .005 and adding any HCTSA feature increased the AUC by less than .005. The performance of birth weight alone is included as well for comparison.

**Table 4.** Comparison of AUC at days 7, 14 and 28 for predicting respiratory outcomes. The HCTSA model used the 3 features sp_walk, sp_mean, and hr_entropy_diff.

Features	Day	Unfavorable	Moderate/severe/death	Severe/death	Death
ih90_dpe	7	0.729	0.715	0.731	0.719
sp_walk	7	0.761	0.724	0.734	0.675
hctsa model	7	0.778	0.754	0.757	0.747
birth weight	7	0.798	0.788	0.786	0.801

ih90_dpe	14	0.791	0.767	0.761	0.741
sp_walk	14	0.797	0.763	0.738	0.689
hctsa model	14	0.800	0.777	0.762	0.765
birth weight	14	0.798	0.787	0.781	0.808

ih90_dpe	28	0.850	0.866	0.843	0.770
sp_walk	28	0.851	0.860	0.834	0.779
hctsa model	28	0.849	0.857	0.831	0.758
birth weight	28	0.792	0.801	0.802	0.849

**Figure 4.**
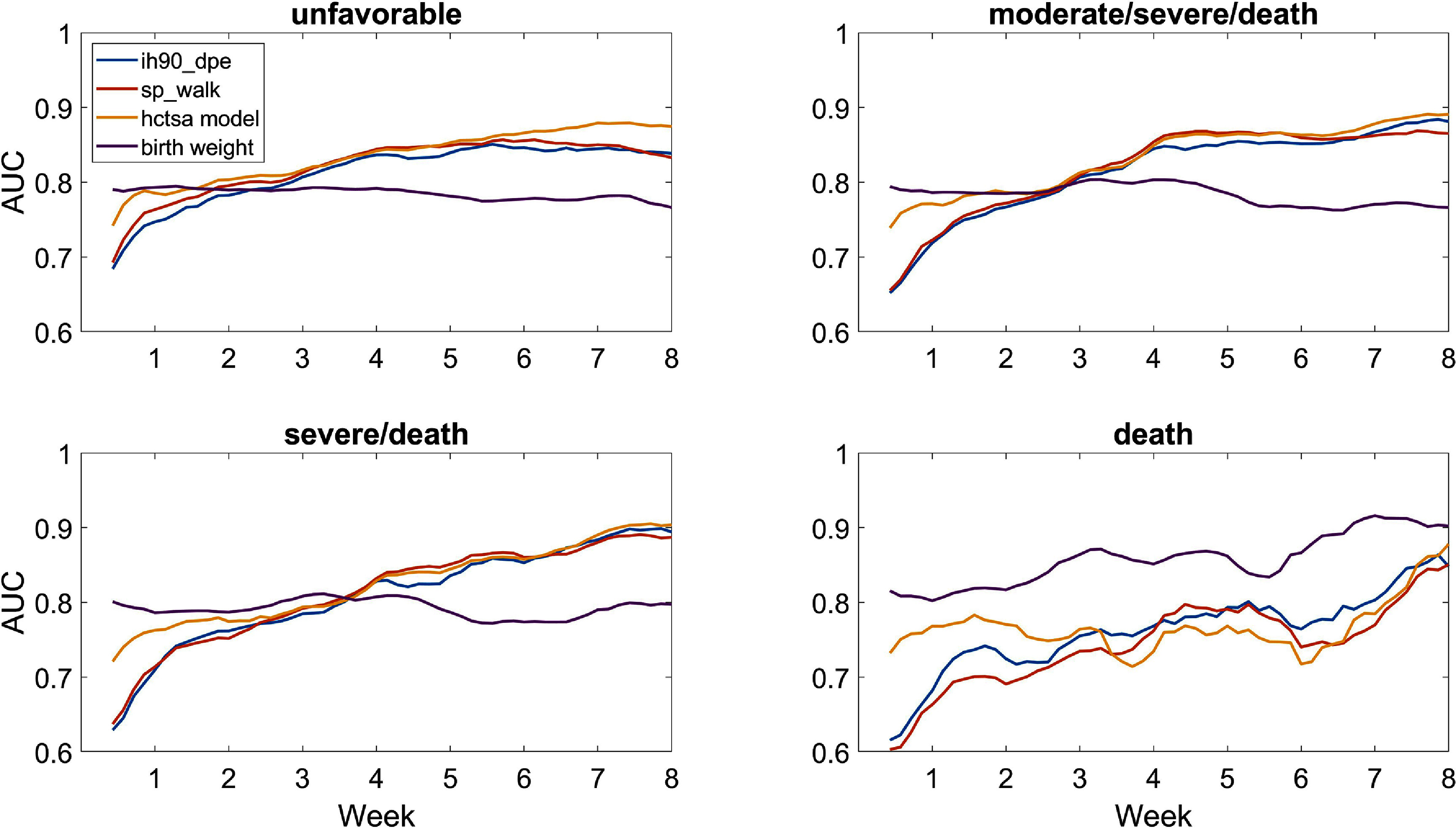
Trajectory of AUC by week of life for predicting respiratory outcomes. Results are averaged over ± 2 days.

**Figure 5.**
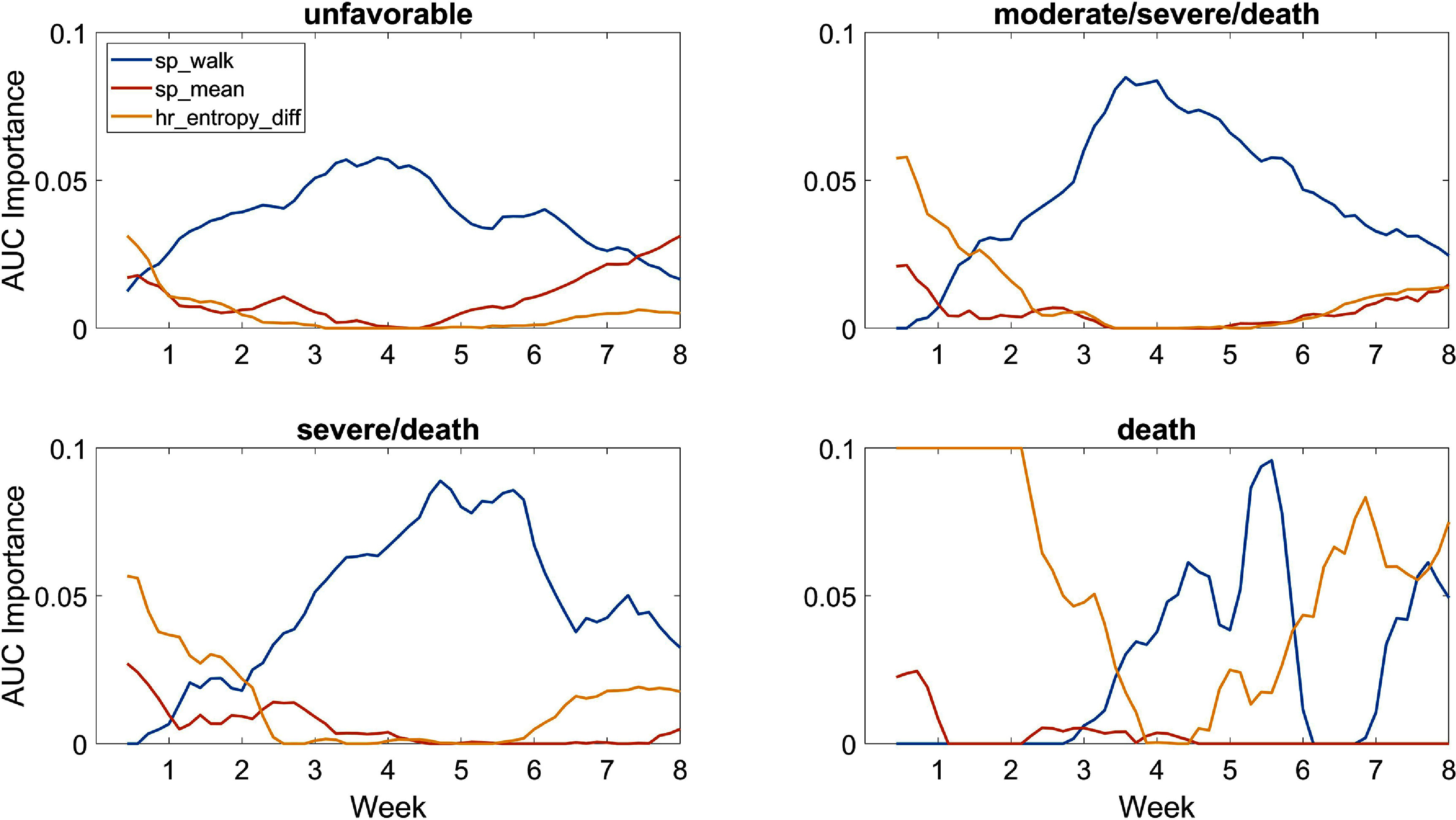
Importance of HCTSA features by week of life measured by drop in AUC when variable is dropped from 3-parameter model. Results are averaged over ±2 days.

The AUC of the models in table 4 were calculated for each respiratory outcome from birth up to 8 weeks of life. Figure 4 shows these performance trajectory curves. The evolving variable importance of each of the 3 features in the HCTSA model is shown in figure 5. All these curves are smoothed by taking average over window of plus or minus two days. Detailed trajectories of the physiologic features including IH events are available for the Pre-Vent cohort (Weese-Mayer 2023).

For unfavorable outcome, the top HCTSA feature slightly outperformed IH90_DPE at day 7 (.761 to .729) but was essentially equivalent at day 28 (.851 to .850). The HCTSA model increased the AUC to .778 at day 7 but did not improve performance at day 28 (.849). Models with new HCTSA features still have a lower AUC than birth weight alone at day 7, but catch up and surpass birth weight by day 14. By week 4 or so, all the physiologic models outperform birth weight for predicting all outcomes except death.

The variable importance of the IH related HCTSA feature sp_walk steadily increases to day of life 28 where it is essentially the only predictor needed. The top HR metric was only an important predictor at early ages which was likely due to the larger proportion of infants whose bad outcome is death by any cause.

## 4. Discussion

This study assessed the ability of using the HCTSA as a tool to identify new types of algorithms for future development of vital sign predictive modeling in the clinical setting using HR and SPO2 as vital signs for predicting respiratory outcomes in preterm infants during the NICU stay. The top new HCTSA features for predicting respiratory outcomes were from a cluster of algorithms associated with the autocorrelation of SPO2 time series and identified low frequency patterns of desaturation as high risk. These features had comparable performance to and were highly correlated with the optimal PreVent physiologic predictor of respiratory morbidity, IH90_DPE, the DPE of intermittent hypoxemia events below 90%. The top HR HCTSA features were symbolic transformation measures that had previously been identified as strong predictors of neonatal mortality.

A simple HCTSA model using 3 top features outperformed IH90_DPE at day of life 7 but was essentially equivalent at day of life 28. HR metrics were only important predictors at early days of life which was likely due to the larger proportion of infants whose outcome was death by any cause. These results demonstrated the utility of the HCTSA approach but also provided additional evidence supporting IH90_DPE as an optimal predictor of respiratory outcomes.

This large multi-center cohort study validated the hypothesis that identifying a small subset of representative algorithms by unsupervised clustering could capture the predictive information in NICU HR and SPO2 data in relation to poor respiratory outcomes. Importantly, this is done without any *a priori* domain knowledge of high risk physiologic signatures for the outcome of interest. This suggests HCTSA is an especially good approach for analyzing time series in settings where there has not been an extensive amount of prior research. As an example of this, HCTSA was recently used to identify a new signature of high HR variability around PMA of 36 weeks as a predictor of cerebral palsy in preterm infants (Letzkus *et al* 2023).

It is worth noting that we are not considering the physiologic measures of apnea and periodic breathing (calculated by BAP using chest impedance waveform) in this analysis. These features are only meaningful for infants not on mechanical ventilation and this represents only about 60% of the days in this study for this cohort. They were also not shown to be major predictors of respiratory outcomes in primary Pre-Vent analysis (Ambalavanan 2023).

In this study, the predictive performance of the features were analyzed at a daily level to be consistent with the PreVent study database and primary analysis. Future work would be to look at higher resolution windows of hourly or even down to individual 10 min records. This would also involve a closer look at individual IH events and their correlation with HCTSA features.

The top HTCSA SPO2 metrics are highly correlated with IH events and provide further data suggesting subtle changes in physiologic status independent of any arbitrary SpO2 threshold may put an infant at risk for poor respiratory outcomes. The new metrics have the advantage of not relying on threshold-based definitions of clinical events and may lead to identifying low or high risk SPO2 patterns even among infants who are in target range. However, the distinct physiology behind these subtle alterations in SpO2 detected by HCTSA has yet to be elucidated.

Although application of HCTSA only marginally improved the predictive model when compared to demographic and clinical risk factors, vital sign features have the advantage of being dynamic and indicate the evolving status of the infant during the NICU stay. In contrast, demographic features are static and fixed at birth. Clinical features like those associated with respiratory support are often in response to changes in the vital signs of the infant and potentially not a direct informative predictor of respiratory outcomes.

As with all metrics based on bedside monitoring data, there are significant practical issues associated with implementation especially for wide-spread clinical use. The algorithms analyzed in this paper in large part came from unsupervised clustering using HR and SPO2 data displayed on bedside monitors every 2 s (Niestroy *et al* 2022). The PreVent sites, however, had a variety of bedside monitors that displayed vital signs either once every second or every 1024 ms. To accommodate for these variations, data was sub-sampled every 2 s at all sites before applying algorithms. For the analysis of HR, one way to minimize variability across sites is to work directly with the interbeat RR intervals derived from ECG waveforms. However, this often involves a significant amount of additional data processing and management that may not be practical. As mentioned previously, SPO2 metrics including IH events can also be sensitive to the averaging time of the pulse oximeter which can vary across vendor and NICU.

## 5. Conclusion

Using a small subset of HCTSA algorithms identified without any *a priori* knowledge of outcome, we were able to achieve comparable performance at day 28 and and better performance at day 7 in predicting respiratory outcomes than simple measures of intermittent hypoxemia event duration derived from extensive clinical experience. These results validate the utility of a representative HCTSA approach but also provides additional evidence supporting intermittent hypoxemia event duration as an optimal predictor of respiratory outcomes. Specifically, we discovered new algorithms associated with the autocorrelation of the SPO2 time series that are highly predictive of respiratory outcomes. While these new metrics are highly correlated with the clinically relevant intermittent hypoxemia events, they remain good candidates to now be included in future work to predict neonatal outcomes using vital sign data.

## Data Availability

The data that support the findings of this study are openly available at the following URL/DOI: https://doi.org/10.18130/V3/SASYHE.

## Appendix Supplemental tables

**Table A1.** HCTSA variable names and Matlab functions available to download from https://github.com/benfulcher/hctsa. The code is well-documented and detailed descriptions of HCTSA functions and input parameters can be obtained by using Matlab Help utility. Features highly predictive of respiratory outcomes (sp_walk, sp_autocorr, hr_entropy_diff, hr_probincreases) are described in detail in Results sections.

Feature	HCTSA variable	Matlab code	Output field
hr_mean	HR.mean	mean(x)	
hr_avgthresh	HR.EX.MovingThreshold.a0.25.b0.1.meanq	EX_MovingThreshold(x,0.25,0.1)	meanq
hr_corrmean	HR.CO.tc3.1..denom	CO_tc3(x,1)	denom
hr_std	HR.std	std(x)	
hr_cv	HR.DN.cv.3	DN_cv(x,3)	
hr_max	HR.Quantile.99	DN_Quantile(x,0.99)	
hr_min	HR.ST.LocalExtrema.n100.minabsmin	ST_LocalExtrema(x,‘n’,100)	minabsmin
hr_kurt	HR.kurt2	kurtosis(x)	
hr_skew	HR.skew2	skewness(x)	
hr_symautocorr	HR.SB.TransitionMatrix23.stdcoveig	SB_TransitionMatrix(x,‘quantile’,2,3)	stdeigcov
hr_surprise	HR.FC.Suprise.mean	FC_Surprise(x)	mean
hr_entropy	HR.SB.MotifThree.quantile.hhhh	SB_MotifThree(x,‘quantile’)	hhhh
hr_wavelet	HR.MF.arfit.sbc.7	MF_arfit(x)	sbc_7
hr_probincreases	HR.SB.MotifTwo.diff.uu	SB_MotifTwo(x,‘diff’)	uu
hr_entropy_diff	HR.SB.MotifThree.diffquant.hhhh	SB_MotifThree(x,‘diffquant’)	hhhh
sp_std	SP.std	std(x)	
sp_mean	SP.mean	mean(x)	
sp_avgthres	SP.EX.MovingThreshold.a0.25.b0.1.meanq	EX_MovingThreshold(x,0.25,0.1)	meanq
sp_corrmean	SP.CO.tc3.1..denom	CO_tc3(x,1)	denom
hr_derivative	HR.SY.StdNthDer.17	SY_StdNthDer(x,17)	
sp_min	SP.ST.LocalExtrema.n100.minabsmin	ST_LocalExtrema(x,‘n’,100)	minabsmin
sp_skew	SP.skew2	skewness(x)	
sp_kurt	SP.kurt2	kurtosis(x)	
sp_mean_trim	SP.DN.RemovePointsmin.0.2.mean	DN_RemovePoints(x,‘min’,0.2)	mean
sp_wavelet	SP.MF.arfit.sbc.7	MF_arfit(x)	sbc_7
sp_symeigen	SP.SB.TransitionMatrix22.mineig	SB_TransitionMatrix(x,‘quantile’,2,2)	mineig
sp_walk	SP.PH.Walkermomentum.2..sw.stdrat	PH_Walker(x,‘momentum’,2)	sw_stdrat
sp_autocorr	SP.AutoCorr.lag.4	CO_AutoCorr(x,4,‘TimeDomainStat’)	
sp_symentropy	SP.SB.MotifThree.diffquant.hhhh	SB_MotifThree(x,‘diffquant’)	hhhh
sp_symbin	SP.SB.TransitionMatrix21.T10	SB_TransitionMatrix(x,‘quantile’,2,1)	T3
sp_symiqr	SP.SB.BinaryMethod.iqr.pstretch1	SB_BinaryStats(x,‘iqr’)	pstretch1

**Table A2.** AUC at day 7 of all individual HR and SPO2 features on increasingly bad respiratory outcomes.

Feature	Cluster	Unfavorable	Moderate/severe/death	Severe/death	Death
hr_mean	1	0.546	0.564	0.576	0.624
hr_corrmean	1	0.547	0.566	0.578	0.626
hr_avgthresh	1	0.554	0.573	0.587	0.638
hr_std	2	0.623	0.643	0.666	0.693
hr_cv	2	0.613	0.629	0.651	0.668
hr_max	3	0.588	0.610	0.628	0.674
hr_min	4	0.490	0.500	0.493	0.517
hr_skew	5	0.550	0.540	0.526	0.492
hr_kurt	5	0.506	0.494	0.532	0.534
hr_symautocorr	6	0.610	0.578	0.555	0.630
hr_surprise	7	0.624	0.625	0.672	0.718
hr_entropy	7	0.675	0.673	0.665	0.736
hr_wavelet	8	0.626	0.635	0.649	0.689
hr_entropy_diff	9	0.689	0.706	0.709	0.765
hr_probincreases	9	0.672	0.688	0.702	0.744
sp_std	10	0.665	0.641	0.598	0.560
sp_mean	11	0.731	0.694	0.692	0.670
sp_corrmean	11	0.731	0.693	0.692	0.669
sp_avgthres	11	0.735	0.699	0.700	0.678
hr_derivative	12	0.601	0.598	0.611	0.651
sp_min	13	0.703	0.687	0.671	0.635
sp_skew	14	0.614	0.587	0.559	0.601
sp_kurt	14	0.698	0.646	0.614	0.608
sp_mean_trim	14	0.725	0.686	0.688	0.667
sp_wavelet	15	0.540	0.503	0.549	0.570
sp_symeigen	16	0.718	0.683	0.648	0.609
sp_autocorr	17	0.740	0.702	0.692	0.645
sp_walk	17	0.761	0.724	0.734	0.675
sp_symentropy	18	0.552	0.509	0.538	0.548
sp_symbin	19	0.595	0.614	0.626	0.618
sp_symiqr	20	0.600	0.626	0.636	0.626
xc_max		0.511	0.559	0.577	0.622
xc_min		0.538	0.571	0.595	0.636
ih90_count		0.696	0.679	0.637	0.604
ih90_dur		0.730	0.715	0.691	0.658
ih90_dpe		0.729	0.715	0.731	0.719
ih80_count		0.652	0.673	0.666	0.678
ih80_dur		0.671	0.692	0.688	0.704
ih80_dpe		0.672	0.685	0.677	0.710
brady_count		0.612	0.631	0.613	0.542
brady_dur		0.595	0.615	0.595	0.517
brady_dpe		0.542	0.584	0.564	0.508

**Table A3.** AUC at day 14 of all individual HR and SPO2 features on increasingly bad respiratory outcomes.

Feature	Cluster	Unfavorable	Moderate/severe/death	Severe/death	Death
hr_mean	1	0.513	0.546	0.569	0.570
hr_corrmean	1	0.513	0.547	0.570	0.571
hr_avgthresh	1	0.522	0.558	0.581	0.587
hr_std	2	0.666	0.694	0.730	0.799
hr_cv	2	0.667	0.687	0.723	0.787
hr_max	3	0.585	0.611	0.635	0.649
hr_min	4	0.522	0.531	0.536	0.569
hr_skew	5	0.615	0.575	0.536	0.498
hr_kurt	5	0.525	0.521	0.575	0.610
hr_symautocorr	6	0.537	0.545	0.537	0.582
hr_surprise	7	0.620	0.632	0.668	0.765
hr_entropy	7	0.647	0.658	0.667	0.747
hr_wavelet	8	0.653	0.672	0.698	0.803
hr_entropy_diff	9	0.704	0.718	0.727	0.807
hr_probincreases	9	0.686	0.704	0.731	0.809
sp_std	10	0.653	0.634	0.560	0.534
sp_mean	11	0.732	0.698	0.686	0.603
sp_corrmean	11	0.731	0.696	0.687	0.603
sp_avgthres	11	0.741	0.708	0.700	0.625
hr_derivative	12	0.629	0.637	0.668	0.745
sp_min	13	0.666	0.636	0.583	0.519
sp_skew	14	0.682	0.664	0.687	0.708
sp_kurt	14	0.750	0.725	0.711	0.668
sp_mean_trim	14	0.720	0.683	0.691	0.631
sp_wavelet	15	0.540	0.559	0.614	0.712
sp_symeigen	16	0.749	0.720	0.657	0.575
sp_autocorr	17	0.782	0.751	0.710	0.659
sp_walk	17	0.797	0.763	0.738	0.689
sp_symentropy	18	0.511	0.516	0.576	0.676
sp_symbin	19	0.672	0.685	0.663	0.694
sp_symiqr	20	0.683	0.695	0.667	0.707
xc_max		0.595	0.628	0.629	0.648
xc_min		0.623	0.661	0.674	0.714
ih90_count		0.663	0.616	0.553	0.549
ih90_dur		0.742	0.699	0.647	0.554
ih90_dpe		0.792	0.767	0.761	0.741
ih80_count		0.675	0.667	0.633	0.596
ih80_dur		0.706	0.700	0.670	0.625
ih80_dpe		0.724	0.734	0.727	0.672
brady_count		0.600	0.614	0.595	0.633
brady_dur		0.586	0.597	0.581	0.635
brady_dpe		0.536	0.549	0.538	0.641

**Table A4.** AUC at day 28 of all individual HR and SPO2 features on increasingly bad respiratory outcomes.

Feature	Cluster	Unfavorable	Moderate/severe/death	Severe/death	Death
hr_mean	1	0.524	0.588	0.608	0.581
hr_corrmean	1	0.525	0.589	0.608	0.580
hr_avgthresh	1	0.533	0.600	0.620	0.589
hr_std	2	0.657	0.679	0.696	0.678
hr_cv	2	0.651	0.655	0.669	0.656
hr_max	3	0.604	0.664	0.681	0.657
hr_min	4	0.515	0.549	0.559	0.569
hr_skew	5	0.679	0.661	0.641	0.584
hr_kurt	5	0.558	0.556	0.541	0.505
hr_symautocorr	6	0.544	0.572	0.552	0.552
hr_surprise	7	0.663	0.673	0.708	0.802
hr_entropy	7	0.635	0.642	0.626	0.673
hr_wavelet	8	0.645	0.679	0.694	0.685
hr_entropy_diff	9	0.688	0.697	0.705	0.719
hr_probincreases	9	0.679	0.702	0.715	0.751
sp_std	10	0.675	0.620	0.588	0.492
sp_mean	11	0.772	0.754	0.748	0.768
sp_corrmean	11	0.769	0.753	0.748	0.770
sp_avgthres	11	0.784	0.771	0.766	0.790
hr_derivative	12	0.612	0.625	0.642	0.378
sp_min	13	0.655	0.595	0.577	0.543
sp_skew	14	0.783	0.779	0.767	0.752
sp_kurt	14	0.817	0.821	0.799	0.745
sp_mean_trim	14	0.765	0.750	0.759	0.790
sp_wavelet	15	0.577	0.652	0.647	0.667
sp_symeigen	16	0.814	0.794	0.750	0.691
sp_autocorr	17	0.841	0.844	0.807	0.728
sp_walk	17	0.851	0.860	0.834	0.779
sp_symentropy	18	0.532	0.543	0.554	0.621
sp_symbin	19	0.681	0.706	0.687	0.673
sp_symiqr	20	0.704	0.728	0.705	0.687
xc_max		0.606	0.652	0.648	0.590
xc_min		0.634	0.673	0.665	0.616
ih90_count		0.691	0.618	0.595	0.580
ih90_dur		0.797	0.760	0.737	0.680
ih90_dpe		0.850	0.866	0.843	0.770
ih80_count		0.714	0.669	0.655	0.604
ih80_dur		0.756	0.727	0.727	0.646
ih80_dpe		0.795	0.798	0.816	0.711
brady_count		0.542	0.541	0.468	0.430
brady_dur		0.475	0.481	0.500	0.429
brady_dpe		0.502	0.510	0.525	0.590

**Table A5.** Correlation at day 7 of all individual HR and SPO2 features with clinical features. BW: birthweight GA: gestational age IMV: invasive mechanical ventilation FIO2: fraction of inspired oxygen.

Variable	BW	GA	IMV	FIO2
hr_mean	0.03	0.04	0.00	−0.09
hr_corrmean	0.04	0.04	−0.01	−0.08
hr_avgthresh	0.06	0.06	−0.02	−0.10
hr_std	0.23	0.23	−0.31	−0.25
hr_cv	0.08	0.08	−0.15	−0.10
hr_max	0.14	0.15	−0.10	−0.17
hr_min	−0.05	−0.01	0.18	0.06
hr_skew	0.11	0.18	0.06	−0.02
hr_kurt	−0.06	−0.12	−0.13	−0.03
hr_symautocorr	−0.30	−0.21	0.24	0.08
hr_surprise	0.36	0.34	−0.33	−0.29
hr_entropy	0.44	0.39	−0.33	−0.22
hr_wavelet	0.30	0.25	−0.33	−0.27
hr_entropy_diff	0.47	0.46	−0.35	−0.28
hr_probincreases	0.47	0.46	−0.38	−0.30
sp_std	−0.26	−0.38	0.36	0.23
sp_mean	0.33	0.40	−0.39	−0.53
sp_corrmean	0.38	0.48	−0.48	−0.58
sp_avgthres	0.33	0.40	−0.40	−0.53
hr_derivative	0.18	0.13	−0.23	−0.17
sp_min	0.34	0.45	−0.40	−0.45
sp_skew	−0.07	−0.17	0.22	0.22
sp_kurt	0.04	0.15	−0.21	−0.17
sp_mean_trim	0.31	0.37	−0.37	−0.52
sp_wavelet	0.01	−0.04	−0.08	0.01
sp_symeigen	−0.30	−0.45	0.46	0.34
sp_autocorr	−0.41	−0.56	0.61	0.41
sp_stdrandwalk	0.48	0.58	−0.66	−0.45
sp_symentropy	0.00	−0.04	−0.12	0.01
sp_symbin	0.22	0.27	−0.37	−0.20
sp_symiqr	0.25	0.30	−0.42	−0.21
xc_max	−0.04	−0.09	−0.10	0.05
xc_min	−0.10	−0.14	−0.01	0.15
ih90_count	−0.29	−0.44	0.35	0.34
ih90_dur	−0.38	−0.53	0.51	0.48
ih90_dpe	−0.42	−0.55	0.66	0.46
ih80_count	−0.25	−0.35	0.37	0.35
ih80_dur	−0.28	−0.35	0.42	0.41
ih80_dpe	−0.35	−0.44	0.51	0.37
brady_count	0.02	0.07	−0.33	−0.13
brady_dur	−0.02	0.04	−0.29	−0.09
brady_dpe	−0.01	0.02	−0.23	−0.08

**Table A6.** AUC at day 7, 14 and day 28 of individual clinical features on increasingly bad respiratory outcomes. BW: birthweight GA: gestational age IMV: invasive mechanical ventilation FIO2: fraction of inspired oxygen.

Feature	Day	Unfavorable	Moderate/severe/death	Severe/death	Death
BW	7	0.798	0.788	0.786	0.801
GA	7	0.765	0.794	0.765	0.782
IMV	7	0.736	0.755	0.745	0.720
FIO2	7	0.763	0.733	0.736	0.696

BW	14	0.787	0.780	0.781	0.808
GA	14	0.770	0.779	0.748	0.780
IMV	14	0.748	0.770	0.752	0.734
FIO2	14	0.797	0.783	0.789	0.765

BA	28	0.792	0.801	0.802	0.849
GA	28	0.776	0.807	0.776	0.833
IMV	28	0.737	0.789	0.777	0.722
FIO2	28	0.829	0.817	0.791	0.754

**Table A7.** Additional time series feature values for SPO2 examples in figure 3.

Panel	Row	Column	sp_walk	sp_autocorr	sp_mean	sp_kurt
A	1	1	1.002	0.962	95.8	−1.13
A	1	2	1.002	0.974	86.5	−1.22
A	1	3	1.001	0.959	75.7	−0.69
A	2	1	1.037	0.463	91.5	−0.24
A	2	2	1.037	0.712	93.4	−0.31
A	2	3	1.037	0.667	99.1	0.46
A	3	1	1.118	0.279	96.0	9.04
A	3	2	1.116	0.271	95.6	0.08
A	3	3	1.131	0.038	95.7	1.52

B	1	1	1.004	0.965	84.4	−0.61
B	1	2	1.001	0.966	92.8	−1.35
B	1	3	1.004	0.964	92.3	−0.74
B	2	1	1.022	0.700	95.0	0.42
B	2	2	1.041	0.698	87.6	−0.29
B	2	3	1.030	0.697	91.2	−0.28
B	3	1	1.100	0.175	97.0	0.30
B	3	2	1.088	0.089	99.8	0.33
B	3	3	1.085	0.187	97.3	−0.53
