# Corrigendum: Highly comparative time series analysis of oxygen saturation and heart rate to predict respiratory outcomes in extremely preterm infants (2024 *Physiol. Meas.* 45 055025)

**DOI:** 10.1088/1361-6579/ad84d4

**Published:** 2024-10-21

**Authors:** Jiaxing Qiu, Juliann M Di Fiore, Narayanan Krishnamurthi, Premananda Indic, John L Carroll, Nelson Claure, James S Kemp, Phyllis A Dennery, Namasivayam Ambalavanan, Debra E Weese-Mayer, Anna Maria Hibbs, Richard J Martin, Eduardo Bancalari, Aaron Hamvas, J Randall Moorman, Douglas E Lake, Pre-Vent Investigators, Katy N Krahn, Amanda M Zimmet, Bradley S Hopkins, Erin K Lonergan, Casey M Rand, Arlene Zadell, Arie Nakhmani, Waldemar A Carlo, Deborah Laney, Colm P Travers, Silvia Vanbuskirk, Carmen D’Ugard, Ana Cecilia Aguilar, Alini Schott, Julie Hoffmann, Laura Linneman

**Affiliations:** 1 Department of Medicine, Division of Cardiology, University of Virginia School of Medicine, Charlottesville, VA, United States of America; 2 Department of Pediatrics, Case Western Reserve University School of Medicine, University Hospitals Rainbow Babies and Children’s Hospital, Cleveland, OH, United States of America; 3 Department of Pediatrics, Division of Autonomic Medicine, Northwestern University Feinberg School of Medicine, Chicago, IL, United States of America; 4 Department of Pediatrics, Division of Neonatology, University of Miami Miller School of Medicine, Miami, FL, United States of America; 5 Department of Pediatrics, Division of Neonatology, University of Alabama at Birmingham, Birmingham, AL, United States of America; 6 Department of Pediatrics, Brown University School of Medicine, Department of Pediatrics, Providence, RI, United States of America; 7 Department of Electrical Engineering, University of Texas at Tyler, Tyler, TX, United States of America; 8 Ann and Robert H. Lurie Children’s Hospital and Northwestern University Department of Pediatrics, Chicago, IL, United States of America; 9 Department of Pediatrics, Division of Pediatric Pulmonology, Washington University School of Medicine, St. Louis, MO, United States of America; 10 Department of Pediatrics, University of Arkansas for Medical Sciences and Arkansas Children’s Hospital, Little Rock, AR, United States of America; 11 University of Virginia Center for Advanced Medical Analytics, Charlottesville, VA, United States of America; 12 Ann and Robert H. Lurie Children’s Hospital of Chicago, and Pediatric Autonomic Medicine, Stanley Manne Children’s Research Institute, Chicago, IL, United States of America; 13 Department of Pediatrics, University Hospitals Cleveland Medical Center, Rainbow Babies and Children’s Hospital, Cleveland, OH, United States of America; 14 Department of Electrical and Computer Engineering, University of Alabama at Birmingham, Birmingham, AL, United States of America; 15 Division of Neonatology, Department of Pediatrics, University of Alabama at Birmingham School of Medicine, Birmingham, AL, United States of America; 16 Division of Neonatology, Department of Pediatrics, University of Miami Miller School of Medicine, Holtz Children’s Hospital—University of Miami/Jackson Memorial Medical Center, Miami, FL, United States of America; 17 Washington University School of Medicine in St. Louis, St. Louis, MO, United States of America

It has come to our attention that the original article did not include the group author name for the ‘Pre-Vent Investigators’ consortium. The collaborator names of the members of the consortium and their institutions are found below:

## 1. Pre-Vent Investigators

Katy N Krahn and Amanda M Zimmet, University of Virginia Center for Advanced Medical Analytics, Charlottesville, VA; Bradley S Hopkins, Erin K Lonergan, Casey M Rand: Ann and Robert H. Lurie Children’s Hospital of Chicago, and Pediatric Autonomic Medicine, Stanley Manne Children’s Research Institute, Chicago, IL; Arlene Zadell, Department of Pediatrics, University Hospitals Cleveland Medical Center, Rainbow Babies and Children’s Hospital, Cleveland, OH; Arie Nakhmani, Department of Electrical and Computer Engineering, University of Alabama at Birmingham, Birmingham, AL; Waldemar A Carlo, Deborah Laney, and Colm P Travers, Division of Neonatology, Department of Pediatrics, University of Alabama at Birmingham School of Medicine, Birmingham, AL; Silvia Vanbuskirk, Carmen D’Ugard, Ana Cecilia Aguilar and Alini Schott, Division of Neonatology, Department of Pediatrics, University of Miami Miller School of Medicine, Holtz Children’s Hospital—University of Miami/Jackson Memorial Medical Center, Miami, FL; and Julie Hoffmann and Laura Linneman, Washington University School of Medicine in St. Louis, St. Louis, MO.

## 2. NIH/NHLBI

Neil Aggarwal, MD: National Institutes of Health, National Heart, Lung and Blood Institute, Division of Lung Diseases, Bethesda, MD. Lawrence Baizer, PhD: National Institutes of Health, National Heart, Lung and Blood Institute, Division of Lung Diseases, Bethesda, MD. Peyvand Ghofrani, MDE: National Institutes of Health, National Heart, Lung and Blood Institute, Division of Lung Diseases, Bethesda, MD. Aaron D Laposky, PhD: National Institutes of Health, National Heart, Lung and Blood Institute, Division of Lung Diseases, Bethesda, MD. Aruna Natarajan MD, PhD: National Institutes of Health, National Heart, Lung and Blood Institute, Division of Lung Diseases, Bethesda, MD. Barry Schmetter, BS: National Institutes of Health, National Heart, Lung and Blood Institute, Division of Lung Diseases, Bethesda, MD.

## 3. OSMB

Estelle B Gauda, MD (Chair): University of Toronto Hospital for Sick Children, Division of Neonatology, Toronto, Ontario. Jonathan M Davis, MD: Tufts Clinical and Translational Science Institute, Division of Newborn, Medicine, Boston, MA. Roberta L Keller, MD: University of California, San Francisco School of Medicine, Department of Pediatrics, San Francisco CA. Robinder G Khemani, MD: Children’s Hospital Los Angeles, Department of Anesthesiology and Critical Care Medicine, Los Angeles, CA. Renee H Moore, PhD: Drexel University Department of Epidemiology and Biostatistics, Philadelphia, PA. Elliott M Weiss, MD, MSME: Seattle Children’s Hospital, Department of Pediatrics, Division of Bioethics, Seattle, WA.

